# Taming the white bear: Lowering reactance pressures enhances thought suppression

**DOI:** 10.1371/journal.pone.0282197

**Published:** 2023-03-02

**Authors:** Matthew Wallaert, Andrew Ward, Traci Mann

**Affiliations:** 1 Department of Psychology, Swarthmore College, Swarthmore, PA, United States of America; 2 Department of Psychology, University of Minnesota, Minneapolis, MN, United States of America; University of New South Wales, AUSTRALIA

## Abstract

Individuals fail to suppress certain thoughts, especially under conditions that tax cognitive resources. We investigated the impact of modifying psychological reactance pressures on thought suppression attempts. Participants were asked to suppress thoughts of a target item under standard experimental conditions or under conditions designed to lower reactance pressures. In the presence of high cognitive load, weakening associated reactance pressures resulted in greater success at suppression. The results suggest that reducing relevant motivational pressures can facilitate thought suppression, even when an individual experiences cognitive limitation.

## Introduction

The desire to suppress thoughts constitutes a basic need long recognized in the psychological literature [1]. Yet individuals sometimes fail at suppression, ending up thinking the very thought they had intended to eschew. In groundbreaking work, Wegner and colleagues demonstrated that asking research participants to “try not to think of a white bear” resulted in subsequent rebound of such thoughts [2]. Wegner et al. explained these findings principally in terms of coming to associate the to-be-suppressed thought with cues both in the immediate environment and in participants’ recent memory—cues used as distracters away from the target item. Those cues subsequently prompted thoughts of the suppression target, particularly after the prohibition to avoid the target had been lifted [3].

Since the publication of the original “white bear” study, additional accounts have been offered for the post-suppression rebound effect. Among them are repeated priming of the unwanted thought [4]; construct accessibility stemming from an uncompleted suppression goal [5]; and inferring a need to express a construct one has found difficult to suppress [6].

Notably, however, in the typical study, participants exhibit occasional failures at avoiding the target thought during the suppression task itself, not just during the post-suppression period [2]. Such *initial* failures at suppression, that is, failures that occur immediately upon the start of the task, would seem to require a different explanation than those described above—one that, for example, does not depend on the existence of past failures. After all, explanations that rely on repeated priming of forbidden material or on the putative need to express a construct one has already found challenging to suppress cannot explain why one *initially* experiences suppression failure.

In 1994 Wegner offered such an explanation [7], alluded to in earlier publications [8, 9], arguing that instructions to suppress a thought set up competing processes: an operating process, which attempts to avoid the relevant target, and a monitoring process, which periodically tests whether the operating process has succeeded. Wegner further argued that the monitoring process is essentially automatic, requiring relatively few cognitive resources. By contrast, the operating process represents a controlled process [10, 11], requiring more resources, and thus is vulnerable to any degradations in cognitive capacity. When one can devote one’s attention to engaging the operating process, that process is likely to be relatively successful, resulting in few suppression failures. By contrast, the imposition of cognitive load is likely to impair the operating process while leaving the monitoring process relatively intact, resulting in the target of the monitoring process, namely, the forbidden thought, emerging into consciousness. Thus, when asked to perform a cognitively demanding task, such as memorizing a nine-digit number [12], an individual who is also trying not to think of a white bear is likely to experience near-immediate failure at the suppression task.

Of course, in their earliest work in this area, Wegner and his colleagues did indeed focus on “ironic rebound” of *formerly* suppressed material that occurred during a post-suppression period [2]. More recently, this post-suppression rebound has also been the focus of work by other researchers, including Förster and Liberman and their colleagues [6, 13–15]. However, later investigations by Wegner and his colleagues in the domain of thought suppression centered on *concurrent* suppression failure [12, 16–18]. Although the model we are proposing and sought to test here may well apply to instances of post-suppression rebound, note that the focus of the studies reported in this article is exclusively on concurrent thought suppression failures under conditions of cognitive load.

Wegner’s cognitive account notwithstanding, are there other ways through which the imposition of cognitive load can impair the ability to suppress thoughts? In particular, might motivational processes also play a key role in many initial suppression failures? And if so, do they suggest ways to improve one’s ability to avoid certain thoughts?

Similar to Wegner [7], we contend that the attempt to suppress thoughts of a stimulus often triggers competing forces that both foster and inhibit suppression success. However, we argue that factors other than the presence of a basic operating and monitoring process can play a role in such suppression—factors that include motivational pressures attributable to psychological reactance.

### Reactance

On the one hand, when asked to suppress a thought, an individual’s conscious attempts not to think of the forbidden stimulus should promote success at the task. On the other hand, instructions that enjoin someone from thinking a particular thought are likely to create strong motivational pressures to do just that, i.e., conjure up the prohibited thought. Such pressures stem from the well-documented phenomenon of psychological reactance, in which an individual is motivated to restore freedom in the face of a barrier or prohibition, resulting in increased desire for a forbidden stimulus [19–23]. These reactance pressures are likely to compel one to think the very thought that one has been directed to avoid.

Theoretically, reactance can be aroused either by attempting to persuade an individual to engage in a certain action (e.g., “You must eat this cookie!”) or not to engage in one (“You must not eat this cookie!”). However, according to the principle of *negative potency* [24, 25], the magnitude of reactance pressures is predicted to be especially strong when an individual is explicitly encouraged to refrain from (rather than engage in) a particular response [20]—precisely the conditions that have been found most likely to result in failure during thought suppression tasks [22, 26, 27]. We tested this prediction in the pilot study reported below.

### Attentional myopia

Strong reactance pressures, such as those postulated to underlie many thought suppression attempts, may be especially difficult to resist when also performing a cognitive task. According to the attentional myopia model [28, 29], in environments featuring competing behavioral pressures, such as the desire to eat high caloric food versus the desire to adhere to one’s (restrictive) diet, limitations placed on attention render it difficult to heed whichever pressure happens to be less salient. In situations involving strong eating cues, limits placed on attentional capacity, such as through the imposition of cognitive load, can make it difficult to attend to relatively weaker diet cues, resulting in increased consumption. By contrast, if diet cues are made disproportionately salient in the environment, then cognitive load should be associated with diminished eating, as the ability to pay attention to relatively less potent consumption cues is impaired. Both effects have been demonstrated in past studies [28, 30]. Parallel attentional narrowing effects produced by alcohol intoxication, in which drunk individuals devote the bulk of attentional resources to salient internal or external cues, have now been shown in a number of investigations [31–33].

The effects of attentional myopia have also been demonstrated in domains other than those involving food consumption. For example, the imposition of cognitive load can result in individuals smoking more or less than they typically would, depending on the relative balance of factors promoting versus inhibiting smoking in the relevant environment [34, 35]. Similarly, attentional narrowing brought about through arousal [36] can be associated with either increased or decreased aggression, again depending on whether cues promoting aggression or inhibiting aggression are more potent [37]. Attentional myopia effects have also been shown in investigations of prosocial behavior [38] and stereotyping [39].

The attentional myopia model holds that self-regulation failure is likely to result whenever pressures promoting disinhibition dominate limited attentional resources, to the neglect of those pressures prompting restraint, that is, inhibiting pressures that could otherwise be heeded in the absence of attentional limitation. Importantly, past research has found that such attention-consuming pressures can be either internal or external, as well as either cognitive or motivational in nature [40]. Indeed, even the most cognitive of cues (e.g., pallid statistics indicating the fat content), let alone more gustatory stimuli (e.g., the good taste of a milk shake), can ultimately be expected to play a motivational role (e.g., influencing consumption patterns) if sufficiently salient under conditions of limited attention [28].

Of equal importance, the cognitive load manipulations we have repeatedly employed in the past (and made use of in the studies reported here) are designed to be sufficiently taxing to limit attention but not so overwhelming so as to distract participants away from the primary task [41]. In other research, we have shown that significantly more demanding cognitive tasks can essentially prevent participants from attending to anything but the load task itself [42].

### Pilot study

In an explicit test of the postulated dominance of “negative” over “positive” reactance, Ward, Chin, DeChiara, and Mann conducted a pilot study in which 181 undergraduate participants were provided with candy and, through random assignment, told either, “Do not eat the candy” (negative reactance condition); “Eat the candy” (positive reactance condition); or given no instruction regarding the candy (no reactance control condition). Responding to an item probing their sense of restricted freedom using a 9-point scale (1 = *not restricted at all*; 9 = *extremely restricted*), relative to responses from participants in the control condition (*M* = 5.80, *SD* = 2.38; *n* = 61), participants in the negative reactance condition, who were restricted from eating the candy (*n* = 59), reported a much greater (and statistically significant) restriction of their freedom (in this case, their freedom to eat the candy; *M* = 6.81, *SD* = 2.06), *t*(118) = 2.48, *p* = .014, *d* = 0.45, 95% CI = [.20, 1.82], than did those in the positive reaction condition (*n* = 61), who were “forced” to consume the candy and asked to indicate how much their freedom to consume food *other* than the candy had been restricted (*M* = 4.28, *SD* = 2.79 vs. *M*_control_ = 4.39, *SD*_control_ = 2.70), *t*(120) = 0.23, *p* > .81. In a second investigation, similar results were obtained using a stimulus more hedonically neutral than candy (and thus presumably less subject to any “self-imposed” dietary restriction, as might have been exhibited by some in the control condition). Using the same 9-point scale, compared to a control condition (*M* = 2.24, *SD* = 187; *n* = 21), participants asked to make up an online password without using an asterisk as one of the characters (*n* = 22) reported much greater (and, again, statistically significant) restriction of their freedom to use the asterisk (*M* = 6.09, *SD* = 2.91), *t*(41) = 5.14, *p* < .001, *d* = 1.57, 95% CI = [2.34, 5.37], than did participants (*n* = 21) instructed to use the asterisk and asked about restriction of their freedom to use characters other than the asterisk (*M* = 2.52, *SD* = 2.38 vs. *M*_control_ = 1.76, *SD*_control_ = 1.26), *t*(40) = 1.30, *p* > .20.

### Present studies

According to the attentional myopia model, to the extent that an instruction to suppress thoughts of a stimulus prompts strong reactance pressures to engage in the prohibited action, those pressures should be particularly difficult to resist when also under high cognitive load. In other words, given the hypothesized presence of strong reactance pressures in many thought suppression studies, participants asked to suppress a specific thought should typically fail to inhibit thoughts of the relevant target stimulus, particularly when they are also experiencing high cognitive load. By contrast, under low cognitive load, it should be possible to devote a significant degree of attention to pressures other than the salient reactance-induced forces that serve to promote thought suppression failure. Accordingly, when attention is not overly taxed, the desire to inhibit thoughts of the target stimulus should not be entirely dominated by reactance-based pressures, and greater success at suppression should be possible.

This analysis further suggests that, even when cognitive load is present, reducing the *potency* of reactance pressures should enhance suppression success. In our primary study reported below, we tested both of the aforementioned propositions, varying both cognitive load levels and the degree of reactance pressures surrounding a thought suppression task. In addition, in order to rule out competing explanations for our hypothesized results, we conducted several additional studies. Those supporting studies and their conclusions are detailed along with the results of the primary study below.

#### Reactance manipulation

Recall we have hypothesized that, when instructed to suppress a mental target, reactance pressures can come to dominate less potent attentional pressures, particularly when attention is limited by high cognitive load, leading to significant thought suppression failure. Specifically, when instructed to “try not to think of a white bear,” the pressure to think of a white bear should be perceived as stronger than the pressure not to. According to the relevant model, however, weakening those strong promoting reactance pressures should reduce monopolization of attentional resources, freeing cognitive capacity to devote to competing restraining pressures and, ultimately, resulting in greater suppression success, even under high cognitive load.

In prior related research [43], participants who were forbidden from consuming a certain food exhibited a relative increase in desire for the food, a result consistent with a reactance-driven motive to reassert freedom [21]. By contrast, participants who were instructed to avoid the food but assured by the experimenter that if they needed to eat it, they should “feel free” to do so, did not show the same reactance-based desire for the forbidden food. In the studies reported below, we adapted this manipulation for use in a thought suppression task. We manipulated reactance pressures by asking participants to try to suppress a thought under typical experimental conditions to produce suppression failure (pioneered by Wegner et al. [2] and extended in later investigations [7]) or to do so under conditions designed to highlight their freedom of choice to suppress. We predicted that participants in the latter condition would experience fewer reactance pressures and would therefore report reduced expenditure of relevant attentional resources.

In a preliminary study, we first investigated the proposed mechanism, whereby strong reactance pressures dominate attention more than weaker ones. Such an approach would enable subsequent observation of performance without demand characteristics prompted by the presences of a process measure [44].

## Preliminary study: Attentional mechanism

Participants in this and all subsequent investigations were treated in accordance with the ethical principles spelled out by the American Psychological Association. The research was approved by the Swarthmore College Institutional Review Board, and written consent was obtained from participants prior to study participation. All participants in every study were conducted through all relevant procedures, and no participant’s data was omitted from the analyses or reported results.

## Methods

A total of 171 undergraduate participants were randomly assigned to one of two conditions. In the standard suppression condition (control condition), they were instructed to “Please try not to think of a white bear.” In the experimental condition (free choice condition), they were instructed to “Please try not to think of a white bear, but of course if you need to, feel free” (in addition to the results of a prior relevant investigation [43], the reactance-lowering effect of this latter instruction was confirmed through ratings detailed below). After engaging in the suppression task for several minutes, all participants were then asked to complete the following dependent measure: “While you were doing the task, to what extent did thoughts of a white bear consume your attention?” (1 = *Didn’t consume my attention at all*; 7 = *Consumed my attention a great deal*).

## Results and discussion

Before turning to the principal result, we report the findings of two supporting investigations in which we relied on judges to confirm the hypothesized strength of the proposed reactance pressures present in our manipulation. Brehm [19] has argued that although individuals may not typically be aware of the presence of reactance pressures (thus limiting the possibility of explicit measurement of the construct), such pressures vary directly with the magnitude of a prohibiting force. Accordingly, in the first supporting investigation, undergraduate judges (*n* = 18) were provided with a written summary of the experimental procedure and asked to rate, in the context of the study, how much pressure they would feel not to think of a white bear under each of the two experimental conditions (with order of question counterbalanced across participants; 1 = *no pressure at all*; 9 = *extreme pressure*). Participants rated the control condition as producing much greater suppression pressure (*M* = 6.28, *SD* = 1.74) than the free choice condition (*M* = 4.61, *SD* = 2.12), paired *t*(17) = 4.30, *p* < .001, within-subjects *d* = 1.01, 95% CI = [.85, 2.49], suggesting, as predicted, stronger reactance pressures in the former than in the latter condition. This result was replicated in a between-subjects investigation, in which participants were either assigned to not think of a white bear (*n* = 32) or instructed to not to think of a white bear but to feel free to do so if they needed to (*n* = 34). Participants in this latter study were then provided with an explicit definition of reactance (“*reactance* describes a state in which people feel their freedom to engage in a certain act is threatened”). Again, using a 9-point scale, those participants in the standard control condition reported significantly greater feelings of reactance (*M* = 6.66, *SD* = 1.62) than did participants in the free choice condition (*M* = 5.27, *SD* = 2.14), *t*(64) = 2.97, *p* = .004, *d* = .73, 95% CI = [.46, 2.33]. By contrast, the two groups did not differ significantly on measures of curiosity or puzzlement, both *t*s < 1, supporting the internal (and discriminant) validity of our reactance manipulation: Telling participants “Try not to think of a white bear, but of course if you need to, feel free” does indeed lower reactance pressures relative to the standard suppression instruction (i.e., “Try not to think of a white bear”).

Having confirmed the hypothesized difference in reactance pressures, turning to the main result, participants assigned to the control condition in the preliminary study (*n* = 89), who had been asked simply to suppress thoughts of a white bear, reported that thoughts of the white bear consumed more of their attention (*M* = 5.46, *SD* = 2.01) than did those participants assigned to the free choice condition (*M* = 4.80, *SD* = 2.06, *n* = 82), *t*(169) = 2.19, *p* = .037, *d* = .32, 95% confidence interval (CI) = [.04, 1.27].

Consistent with the predictions of the attentional myopia model, a manipulation designed to weaken putative reactance pressures by highlighting participants’ freedom of choice resulted in less attention devoted to the forbidden thought. According to the model, this freeing of attentional resources away from the suppression target should render the suppression processes easier, resulting in greater success at avoiding the forbidden thought. In other words, consistent with theorizing by Steele and Josephs [45], by lowering the putative burden placed on attention by strong reactance pressures, participants should be able to allocate more attention toward engaging in successful distraction away from the suppression target, even when under conditions of attentional limitation.

In our primary study, we therefore tested the prediction that weakening reactance pressures would indeed result in greater suppression success, even under conditions of cognitive load. Importantly, in the absence of high cognitive load, little difference in suppression success was expected, as even strong reactance pressures can be countered by opposing suppression efforts if cognitive resources are not overtaxed. This prediction, with only small observed differences between suppression instruction conditions under low load but large differences expected under high load, is bolstered by the fact that that precise pattern has been observed in prior studies exploring behaviors other than thought suppression [28, 32].

In accompanying investigations, we also provided data that served to rule out alternative accounts for the relevant phenomenon.

## Primary study

In this study, we once again manipulated reactance pressures by asking participants to try to suppress a thought under typical experimental conditions or to do so under conditions designed to highlight their freedom of choice to do so. We predicted that participants in the latter condition would experience reduced reactance pressures and, consequently, enhanced suppression success, even when under the attentional limiting effects of cognitive load.

## Methods

A total of 108 undergraduates participated in exchange for introductory psychology course credit. This sample size was larger by almost 30% than samples employed in seven of eight past relevant studies investigating thought suppression [2, 9, 12, 17] and simply made use of every participant available in the relevant subject pool, without any exclusions or premature termination of the study.

All participants were asked to complete a standard thought suppression task that has been used repeatedly by other researchers in published studies [46–48]. Each participant received a sheet of paper with the heading “My Thoughts” and a column on the right side with a sample check mark at the top. Participants were told that they would be listing their thoughts for five minutes, and in the control condition they were further instructed to “try not to think of a white bear.” By contrast, in the free choice condition, participants were told, “Try not to think of a white bear, but of course if you need to, feel free.” All participants were asked to put a check mark in the column on the right side of the sheet each time they thought of a white bear.

Participants were also asked to memorize either a one-digit number (low cognitive-load condition) or a nine-digit number (high cognitive-load condition). The experimenter then reminded participants one final time not to think of the relevant stimulus, instructed them to begin listing their thoughts without writing down the number they were to memorize, and left the room for five minutes, returning to collect the thought-listing sheet.

## Results and discussion

**Fig 1:**
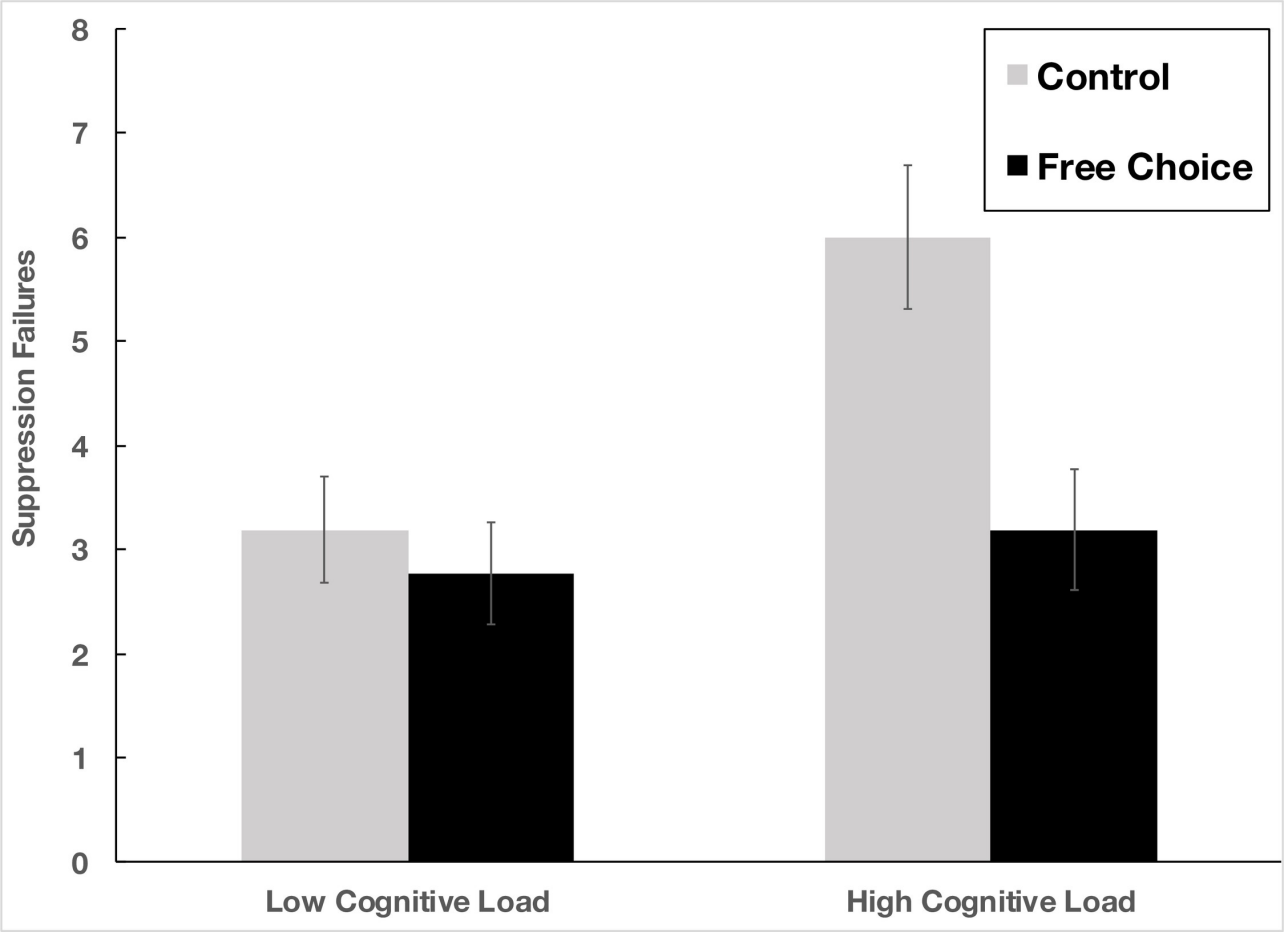
Mean level of thought suppression failure as a function of cognitive load and suppression instruction. Error bars indicate standard errors.

A 2 (control vs. free choice) x 2 (low vs. high cognitive load) analysis of variance (ANOVA) revealed significant main effects of both task instruction, *F*(1, 104) = 7.87, *p* = .006, and cognitive load, (also) *F*(1, 104) = 7.87, *p* = .006, along with the predicted interaction between the two factors, *F*(1, 104) = 4.34, *p* = .040, η_*p*_^2^ = .04 (Fig 1). Averaging across cognitive load conditions, participants asked to suppress white bear thoughts in the high reactance (standard control: “Try not to think of a white bear”) conditions indicated greater suppression failures (*M* = 4.62, *SD* = 3.47) than did those asked to suppress thoughts under conditions designed to reduce reactance pressures (“Try not to think of a white bear, but if you need to, feel free”; *M* = 2.98, *SD* = 2.74), *t*(106) = 2.71, *p* = .008, *d* = .52, 95% CI = [.44, 2.83]. Moreover, averaging across reactance conditions, those asked to perform suppression under conditions of low cognitive load were more successful (*M* = 2.98, *SD* = 2.56) than those asked to perform under conditions of high cognitive load (*M* = 4.62, *SD* = 3.60), (also) *t*(106) = 2.71, *p* = .008, *d* = .53, 95% CI = [.44, 2.83]. Furthermore, as revealed by the significant interaction, under conditions of low load, participants asked to suppress thoughts of a white bear under standard conditions did not perform significantly worse (*M* = 3.19, *SD* = 2.65) than did those asked to suppress thoughts under free choice conditions (*M* = 2.77, *SD* = 2.50), *t*(51) = 0.59, *p* > .55, *d* = .16, 95% CI = [-1.01, 1.84]. By contrast, under conditions of high load, those asked to suppress white bear thoughts in the control condition failed at a significantly higher rate (*M* = 6.00, *SD* = 3.65) than did those in the free choice condition (*M* = 3.19, *SD* = 2.99), *t*(53) = 3.12, *p* = .003, *d* = .84, 95% CI = [1.01, 4.62]. Put another way, consistent with our hypothesis, although participants’ ability to suppress thoughts of a white bear under standard conditions was hampered by high cognitive load, *t*(53) = 3.26, *p* = .002, *d* = .88, 95% CI = [1.09, 4.55] (thus replicating the pattern of results reported by Wegner [7]), under conditions designed to lower reactance pressures, the imposition of high cognitive load did not significantly hinder participants’ ability to suppress thoughts, *t*(51) = 0.55, *p* > .58, *d* = .15, 95 CI = [-1.94, 1.11].

An analysis of high cognitive-load condition participants’ success at recalling the 9-digit number revealed no difference between the control and free choice conditions, *t* < 1. Moreover, an analysis including a factor accounting for whether or not participants correctly recalled the relevant number revealed no alteration of the significance of the findings (indeed, this analysis slightly strengthened the statistical significance of the primary finding, *F*(1, 102) = 5.45, *p* = .022). Similarly, analyses that relied on “simple effects” derived from the overall mean square error of the relevant ANOVA, rather than independent sample *t*-tests, mirrored the significance of the primary effects we reported: In the control condition, participants under high cognitive load evidenced much greater suppression failure than did those under low load, *F*(1, 104) = 12.17, *p* = .001, whereas in the free choice condition, the two cognitive load conditions did not differ, *F*(1, 104) = 0.26, *p* > .61.

In sum, by reducing reactance pressures and essentially giving participants permission to fail at the high-load suppression task, we heightened their success, bringing their performance in line with those not under load.

### Ruling out alternatives: Effort ratings

The possibility that the reactance-lowering instructions might have completely forestalled participants’ efforts at suppression also warranted investigation, as withholding *all* effort at mental control actually may prevent suppression failure [16]. To address this potential confound, we ran an additional sample of 40 undergraduates through the basic thought suppression task. After the five-minute suppression period had ended, participants randomly assigned to the standard suppression condition (*n* = 20) and the free choice condition (*n* = 20) were asked to indicate, by means of a 9-point scale, how hard they had tried not to think of a white bear (1 = *Didn’t try at all*; 9 = *Tried extremely hard*). The results revealed little difference in the mean degree of effort expended by participants in the standard (*M* = 5.20, *SD* = 2.02) vs. free choice (*M* = 5.10, *SD* = 2.13) condition, *t*(38) = 0.15, *p* > .87, suggesting that the manipulation of reactance-relevant instructions did not prevent participants from trying to suppress. A second study involving a much larger sample of participants (*n* = 339) did reveal a significant difference in effort expended by participants in the standard (*M* = 5.23, *SD* = 2.11) vs. free choice (*M* = 4.53, *SD* = 2.23) condition, *t*(337) = 2.97, *p* = .003, *d* = .32, 95% CI = [.24, 1.16]. However, even in this investigation, participants in the free choice condition reported substantial effort expended, rather than a withholding of all effort. Moreover, given the hypothesized greater ease of suppression in the free choice condition, it is not surprising that that condition demanded less effort from participants. The important point is that the results suggest that *both* the standard and free choice conditions required effort and thus did not free participants from the responsibility of engaging in suppression.

#### Ruling out alternatives: Inference ratings

One additional alternative explanation merited detailed consideration in a supporting investigation. Förster and Liberman [14] have speculated that participants might infer from the experimenter’s suppression instructions that they are going to encounter difficulty with the task, perhaps motivating them to think the forbidden thought. As they argue, “[A] participant in a suppression experiment might think, ‘Why would the experimenter ask me not to think of white bears unless he or she thought that I was about to do that? Probably, then, in this experiment I will feel compelled to think of white bears’” (p. 389). To the extent that participants were especially likely to harbor such a conviction in the control condition of our study (as opposed to the free choice condition), this belief would potentially offer an alternative explanation for our results.

Note that this alternative (if valid) and our account would both seem to suggest greater compulsion to use the suppressed construct in the control (“try not to think of a white bear”) rather than the free choice condition (“try not to think of a white bear, but of course if you need to, feel free”). However, only Förster and Liberman’s account suggests that the reason for this difference in putative suppression failure rates lies in a difference in beliefs imputed to the experimenter across the two conditions. Accordingly, we exposed an additional 78 undergraduate participant judges to one of the two suppression instructions and then asked them, “Based solely on those instructions, to what extent did the experimenter think the participant would in fact be motivated to think of a white bear?” (1 = *not at all*; 9 = *a great deal*). Although there was no significant difference between the two conditions, *t*(76) = 1.64, *p* > .10, participants who read the free choice condition instructions reported that they believed the experimenter was *more* likely to think the participants would be motivated to think of a white bear (*n* = 39, *M* = 7.03, *SD* = 1.95) than did participants exposed to the standard suppression instructions (*n* = 39; *M* = 6.23, *SD* = 2.31). As Förster and Liberman’s [14] potential alternative explanation would appear to require that participants in the free choice condition should have thought that the experimenter was *less*—not more—likely to think that they would be motivated to think of a white bear, such a finding suggests that, given the results we obtained (i.e., greater suppression failure in the control condition than in the free choice condition), they could not be accounted for by this alternative explanation.

In sum, our supporting investigations indicated that the results we obtained in the primary study could be attributed to differences in reactance pressures, whose varying strength was confirmed by raters, and not to either a withholding of effort or inferences about the experimenter’s instructions in the reactance-lowering conditions.

## General discussion

Consistent with a significant number of studies conducted by Wegner and colleagues in an investigation of what he termed “ironic process theory” [7], using standard suppression instructions, we replicated the failure to suppress a target thought under conditions of high cognitive load. Also consistent with Wegner’s theorizing, participants who were not under such load were much more successful at the suppression task. They were evidently relatively capable of countering potent pressures to think of the relevant forbidden stimulus, and thus they ended up producing fewer suppression failures. As Wegner had shown, the introduction of a 9-digit memorization task into the standard suppression procedure did indeed result in a heightened level of suppression failure, in which promoting pressures evidently dominated inhibiting pressures to a degree not observed in the absence of such cognitive load.

However, in novel findings not previously reported and not easily assimilated by existing ironic process accounts, a manipulation intended to reduce a potent motivational force, i.e., reactance pressures, effectively eliminated the documented impairment produced by high cognitive load on participants’ suppression abilities. According to the attentional myopia model, and bolstered by the findings of the preliminary study, a manipulation that emphasized participants’ free choice to suppress lowered reactance pressures and freed up cognitive resources. Those resources could then evidently be devoted to successful suppression, even under conditions of limited attention.

Of course, there are limitations associated with our reported results. In particular, our studies made use of undergraduate psychology students as participants, subject to the familiar criticism of relying largely on “WEIRD” samples [49], and no attempt was made to investigate the generalizability of our findings beyond this respondent pool. We also did not ask participants to report their gender or ethnicity, and therefore it is not possible to test for moderation by these individual difference variables, a regrettable oversight that is important for us to correct in future work [50]. In addition, we did not conduct a priori power analyses, knowing that we would instead use every participant available to us in our college participant pool and that our sample sizes would at least exceed those in past similar research. However, some of the sample sizes and effects reported here were not especially large.

### Other suppression domains

Those potential limitations notwithstanding, our results suggest that certain stimuli should be relatively straightforward to suppress, even under conditions of cognitive load, if doing so does not arouse strong reactance pressures. One such category of “low reactance” responses includes behaviors that, though involving suppression, are freely chosen from the outset because they are consistent with the individual’s current goals, as opposed to being imposed by an experimenter who suddenly expects participants to suppress thoughts of a “white bear” (a highly unusual request in the relevant context). In short, in situations in which an instruction to engage in suppression is consistent with the individual’s own goals (i.e., “ego-syntonic” in terms expressed by Freud [51]), strong reactance pressures and accompanying suppression failure are not predicted to occur.

### Past considerations of reactance and thought suppression

In past work, a number of researchers have entertained—and then dismissed—reactance as a mechanism underlying thought suppression failure [14]. For example, Wegner et al. raised psychological reactance as an alternative explanation for their thought suppression “rebound” findings [2]. However, they rejected such a proposition without first testing it, asking, Why should instructing participants *not* to think of a white bear produce a greater level of task failure than instructing participants *to* think of a white bear? (a difference the authors had documented). As they stated, “The difficulty with this [reactance] interpretation comes when we try to understand why a negative injunction should create more reactance than a positive one” (p. 8). Wegner [7] also argued that reactance can be distinguished from his ironic process theory account of suppression failure because the former state “can come and go without the occurrence of intentional mental control” (p. 39). We do not dispute that explanations invoking reactance can apply to situations other than those involving thought suppression [23]).

The aforementioned negative potency findings [24] and results of our pilot investigations (see above) notwithstanding, there may be a simple reason why a negative injunction regarding a forbidden thought produces more reactance than a positive directive to think a particular thought. Wegner himself offered such a reason, invoking an elegant distinction between feature negative and feature positive searches [52]. According to Wegner, when an individual is instructed not to think a certain thought (e.g., “try not to think of a white bear”), the monitoring process—that essentially automatic cognitive process that searches for instances of failure at the task—is given a relatively simple task: search for instances in which the thought arises (e.g., notice every instance in which a white bear thought emerges into consciousness)—a task known as a feature positive search [7]. By contrast, when an individual is instructed to concentrate on a particular thought (e.g., “try to think of a white bear”), the monitoring process is presented with a more difficult challenge in its appointed task to search for failures to complete the task. It must search for instances in which the thought does not arise (e.g., notice every thought that does not in any way constitute a white bear). This type of search, known as a feature negative search, is more difficult because the monitoring process is not presented with as clear a target to indicate failure has occurred (i.e., what precisely constitutes a “not white bear” thought?).

We believe that the distinction between feature positive and feature negative searches can also explain why negative reactance (i.e., the motivational pressure following an instruction *not* to engage in a certain behavior) is more powerful than positive reactance (i.e., the motivational pressure following an instruction *to* engage in a certain behavior). To the extent that reactance motivates an individual to engage in a response opposing the pertinent command, it is not difficult to understand why instructing someone not to think of a white bear is more reactance-arousing that instructing someone to think of a white bear. In the former case, the reactance-prompted opposing response is clear (“think of a white bear”); in the latter case, the response is less clear (“think of something that is not in any way a white bear”). As a result, negative reactance is predicted to create more of a preoccupation with an opposing state than is positive reactance. In short, the same feature positive vs. feature negative search processes that Wegner introduced to explain the greater failure rate of thought suppression over concentration can also explain why negative reactance is stronger than positive reactance.

### Dispositional reactance

Although some theoretical accounts have suggested—and then summarily rejected without explicit experimental manipulation—a reactance explanation for thought suppression failure, past research has attempted to explore the role of *dispositional* reactance in the relevant phenomenon. Kelly and Nauta, for example, measured participants’ personal tendencies to experience reactance and found that those higher in the relevant trait were more likely to experience intrusions of a thought they had been instructed to suppress [22]. However, the reported results failed to reach conventional levels of statistical significance. Indeed, the basic thought suppression vs. thought expression instruction employed in the study failed to produce a difference in intrusions that was statistically significant at conventional levels.

One possibility for these failures may involve the fact that participants in the study were not asked to suppress an unusual thought but, rather, were instructed to suppress their “most frequently occurring intrusive thought.” As was found by Kelly and Kahn [53], asking participants not to think about a thought that they have previously encountered many times (one that they may be well practiced at avoiding) may result in greater task success than when an individual is confronted with a novel target for suppression.

Given these past studies, it is perhaps surprising that no previous attempt has been made to explicitly manipulate, rather than measure, reactance and observe the consequences for thought suppression, as was done here. The results of this investigation suggest for the very first time that direct alteration of reactance pressures can impact thought suppression success, just as a previous study found that food desires could be influenced through a similar reactance manipulation [43].

### Implications for mental control

Inherent in any situation requiring the exercise of self-control is conflict [54]. In the studies reported here, participants faced a conflict between trying to suppress a target thought while contending with potent forces prompting them to think the forbidden thought. These findings suggest clinical implications. Wegner [16] has argued that failures in mental control may underlie certain forms of psychopathology, such as anxiety disorders and depression [55]. These failures can be exacerbated by cognitive demands, leading to rumination and intensification of negative states [18]. Our results suggest that to the extent that individuals can give themselves permission to think forbidden thoughts, then even when cognitive load is present, it may be possible to suppress such thoughts to a degree that minimizes ensuing pathology [16, 56, 57].

In some ways, the reactance-lowering approach described here also mirrors similar recent efforts in clinical domains that encourage individuals to free themselves from crippling guilt or anxiety and simply accept their current state without judgment [58]. Such mindfulness-based approaches have attracted the attention of many clinical researchers in recent years. In the words of one review of this approach [59], “The goal is to end the struggle with unwanted thoughts and feelings without attempting to change or eliminate them” (p. 5). One intriguing outcome of the approach described in the studies reported here is that, in essentially being granted “permission” to think a forbidden thought even while under cognitive load, that thought was ultimately more effectively eliminated than when given a more explicit prohibition against the relevant cognition.

## Conclusion

Suppression failure under cognitive load presented itself as an ideal target for investigation and explanation using the attentional myopia model. We do not mean to imply, however, that alternative explanations for the same phenomenon enjoy no validity. Indeed, we agree with Wegner and Schneider that thought suppression failure under cognitive load may be amenable to several theoretical accounts [60], all of which are consistent with the available data (including more recent neurological findings [61]). Although the analysis reported here suggests a potential alternative explanation for a host of relevant findings, we see its real value in providing a distinct strategy for enhancing success at thought suppression under load. Of course, the best approach to suppression may be not to attempt it at all [16, 62], but barring that, reducing reactance may offer a path to tamer thoughts, whether they be about white bears or anything else.
